# CH_3_NH_3_PbX_3_ (X = I, Br) encapsulated in silicon carbide/carbon nanotube as advanced diodes

**DOI:** 10.1038/s41598-018-33668-5

**Published:** 2018-10-12

**Authors:** Lishu Zhang, Xinyue Dai, Tao Li, Jie Li, Hui Li

**Affiliations:** Key Laboratory for Liquid-Solid Structural Evolution and Processing of Materials, Ministry of Education, Shandong University, Jinan, 250061 People’s Republic of China

## Abstract

We employ first-principles density functional theory (DFT) calculations to study CH_3_NH_3_PbX_3_ (X = I, Br) and its encapsulation into the silicon carbide nanotube and carbon nanotube (CNT). Our results indicate that these devices show diode behaviors which act on negative bias voltage but do not work under positive voltage. When they are encapsulated into SiC nanotube and CNT, their electronic properties would be changed, especially, electric currents mainly exist at positive bias region. Corresponding transmission spectra and density of states are provided to interpret the transport mechanism of the CH_3_NH_3_PbX_3_ (X = I, Br) as a diode. These findings open a new door to microelectronics and integrated circuit components, providing theoretical foundation for innovation of the new generation of electronic materials.

## Introduction

Because of its rapid increment in the power conversion efficiencies as well as its potential applications of high-performance photovoltaic devices, the hybrid lead trihalide perovskite attracted world attention widely. A race has started on both the development of new synthesis approaches and the research on fascinating properties of these materials^1–7^. With the former active research, the usage of halide perovskites has expanded not only in photovoltaic devices but also in other equally important applications such as photodetectors^7^, lasers^8^, and light emitting diodes (LEDs)^9^. Furthermore, many excellent experimental works have been done to research CH_3_NH_3_PbX_3_ as photovoltaic material, where X = I, Br, and Cl. And most important experimental results were obtained in the preliminary phase of study for CH_3_NH_3_PbX_3_ as photovoltaic material^5,10–14^. Prior to that, the search for CH_3_NH_3_PbX_3_ bulk materials includes nuclear magnetic resonance (NMR) spectroscopy^15^, X-ray diffraction^16^, ptical characterization techniques^17,18^, dielectric and millimetre wave measurements^19^, calorimetry^20^, which is a series of quite a few fundamental problems. Recently, many more advanced experimental methods have emerged, such as thermally stimulated current (TSC) measurements which is used to study point-defect and trap in CH_3_NH_3_PbI_3_^21^. On the other hand, the former researchers have fabricated hybrid perovskites as single crystals,, thin films, or nanocrystals (NCs), which have not noly novel optical properties but also excellent electrical properties. At the nanoscale, especially, they are advanced and superior in exhibit technology because of its higher photoluminescence quantum yield (PLQY)^22^ and quantum confinement effect. Up to now, much work has been done to fabricate CH_3_NH_3_PbX_3_ at the nanoscale successfully. For instance, Vybornyi *et al*.^23^ have verified cubic shapes for CH_3_NH_3_PbI_3_ nanocrystals (NCs) and nanoplatelets for CH_3_NH_3_PbBr_3_. And Shamsi *et al*.^24^ have reported that halide perovskites employing N-methyl formamide of methylammonium (MA). The cubic structure for MAPbCl_3_ and MAPbI_3_ NCs as well as the tetragonal phase for the MAPbI_3_ NCs was confirmed by XRD patterns of the different NC samples.

A great deal of operating light emitting devices that was established upon MAPbX_3_ have indeed been demonstrated^9,25–29^, and the recent development of organic-inorganic halide perovskite materials including MAPbI_3_, the light harvesters in solid-state sensitized solar cells, has accomplished with a significant efficiency values exceeding 20%^30^. In addition, the structural properties of these hybrid lead trihalide perovskites, which are arranged in parallel, have also been studied^31^. However there is not enough study on their electrical transport properties and its current-voltage characteristic. Moreover, their potential application in the logic circuit such as diodes has not been reported so far. We also suppose the electronic behavior of MAPbX_3_ would change with carbon nanotube or silicon carbide nanotube encapsulated. How do these perovskite devices exhibit unidirectional penetrability in current-voltage characteristics? Can large influence occur in the current-voltage characteristics of these hybrid lead trihalide perovskite devices with carbon nanotube and silicon carbide nanotube? All the questions above, which are critical for perovskite electronics, need to be further throughly studied. The goal of this work is to solve these two issues.

To this end, we present a first-principles DFT study of MAPbX_3_ (X = I, Br) and their encapsulation in silicon carbide/carbon nanotube as a function of composition. By adopting computation in this way, we demonstrate sensitivity to electronic properties information on the microscopic scale, hence, it can connect microscopic scale structure to novel electronic properties determined by this work, informing new design for novel efficient and stable diodes which can be employed in confidential-needing aspect.

## Results

**Figure 1:**
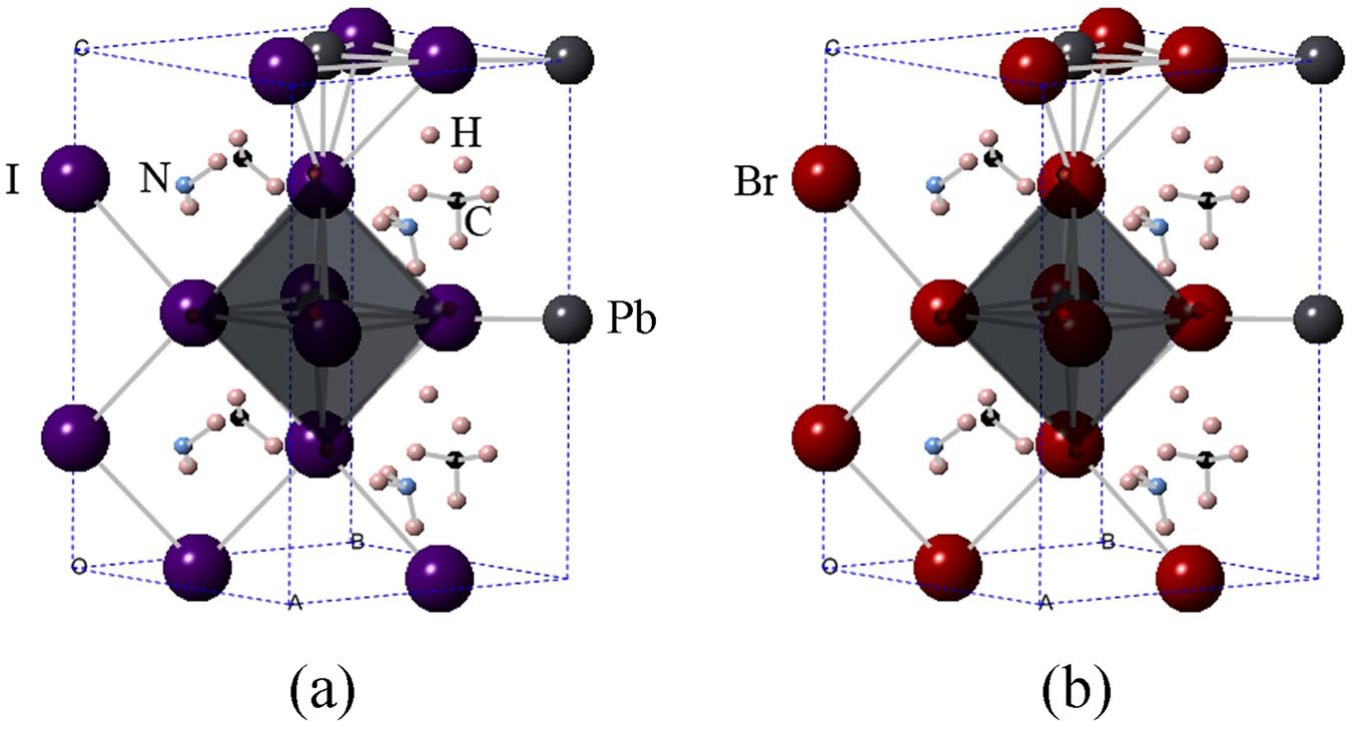
Atomic structures of (**a**) CH_3_NH_3_PbI_3_, (**b**) CH_3_NH_3_PbBr_3_. Key: grey-Pb, purple-I, red-Br, blue-N, black-C, and pink-H.

**Figure 2:**
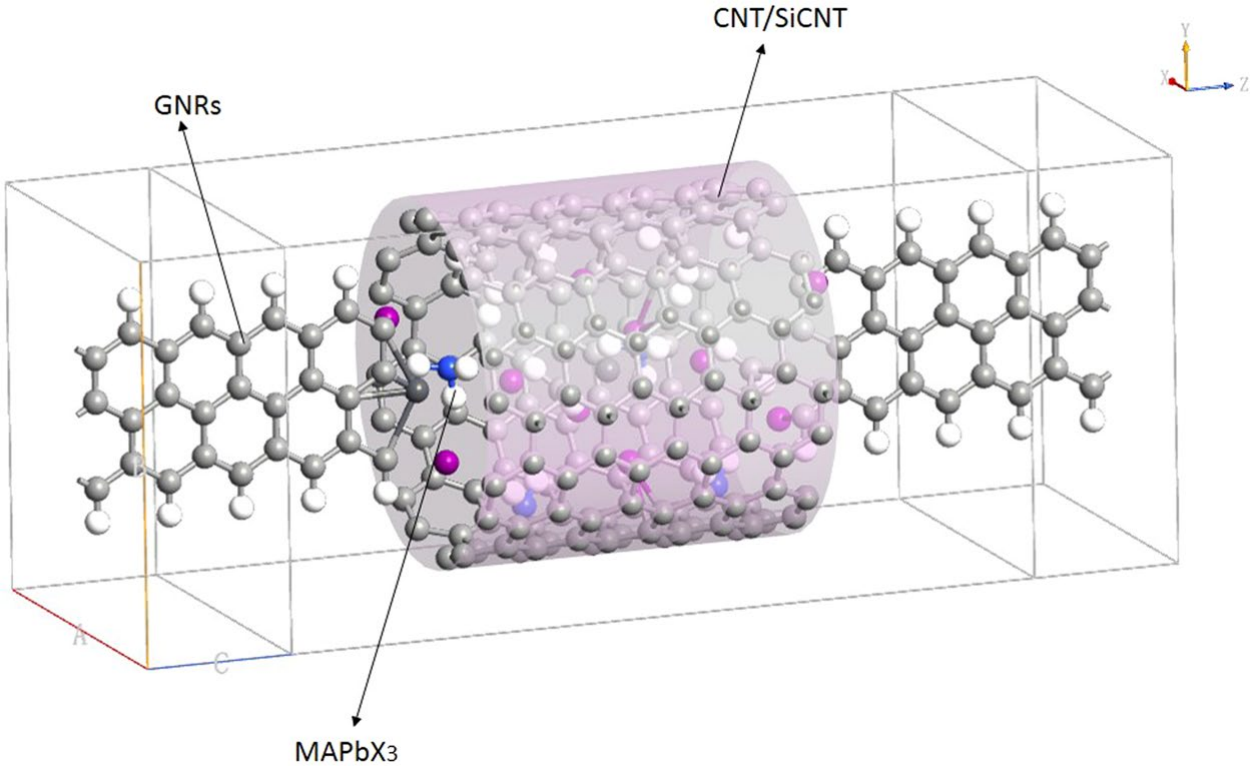
Schematics of CH_3_NH_3_PbX_3_ (X = I, Br) encapsulated in silicon carbide/carbon nanotube.

Our calculations assess the current sensitivity to halide identity and the influence of the encapsulation of nanotubes. Figure 1 exhibits the atomic structures of CH_3_NH_3_PbI_3_ and CH_3_NH_3_PbBr_3_ designed by Feng and Xiao^32^. Analogous to CH_3_NH_3_PbI_3_, perovskite CH_3_NH_3_PbBr_3_ has the same lattice structure, in which Br and I have the same position. Although peroviskites possess several advantages, they are not stable in presence of moisture and heat. In consideration of the rapid moisture-induced degradation of the system, the encapsulation sealing will be important for perovskites^33^. It has been reported that nanotube in solar cells based on perovskites is better in electron transport and recombination behaviors than traditional films^34^. And the single-walled carbon nanotube is found to create an additional barrier to degradation^35^. All these results suggest the encapsulation plays a key role in determining the stability. Many experiments of carbon nanotubes have been done^36–38^, which make the encapulation of nanotubes possible. Inspired by the above studies, we here insert the molecules CH_3_NH_3_PbI_3_ and CH_3_NH_3_PbBr_3_ into the silicon carbide nanotube and carbon nanotube to explore their electronic properties and potential applications in electronic circuits, as shown in Fig. 2.

**Figure 3:**
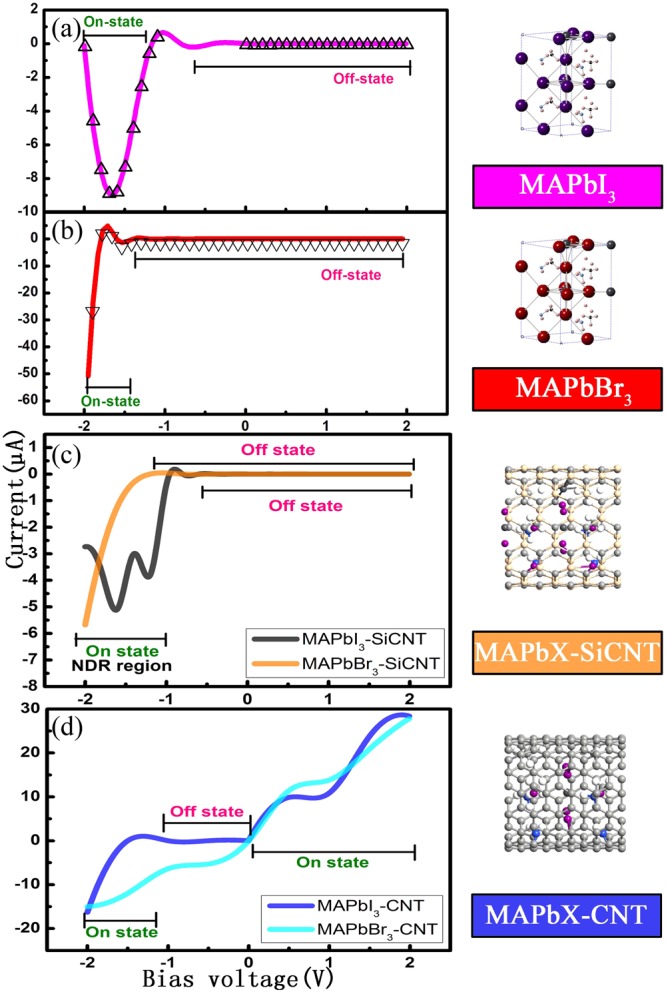
I–V characteristic curves of CH_3_NH_3_PbI_3_ and CH_3_NH_3_PbBr_3_ and their encapsulation with silicon carbide/carbon nanotube.

Figure 3 depicts the current−voltage characteristics of the six models studied in this work. I–V characteristics have been measured by Sarswat *et al*.^39^ and Tang *et al*.^40^ showing the memory effect and negative differential resistance (NDR) effect. And these curves they obtained have two characteristics. One is a local current maximum (when V = V_max_), and then the NDR effect appear. Another is the dual states when V < V_max_, where the on state is the situation when the first time the device reach the V_max_. Before quickly returning to zero, restore the off state by accessing a voltage that exceeds the NDR region. Although the NDR effect is also appear in our study, there are some differences. It is not performed voltage sweep like Sarswat *et al*.^39^ and Tang *et al*.^40^. And no memory effect display. The only I–V characteristic of one model is calculated based on the corresponding transmission spectra at different bias voltages one by one. Here, for simplicity, we regard the region when no current appears (I = 0A) as off state and the region when current has value (I ≠ 0A) as on state. We can see from Fig. 3(a) that MAPbI_3_ model has unidirection continuity of the diode, showing on-state in the negative bias and off-state in the positive range. The reverse recovery (RR) phenomenon occurs when a negative voltage is applied to a certain value across the MAPbI_3_ device. When the stage of the reverse current starts to build up, the current would reaches its peak value and then drop back to the beginning state (off state). What is noteworthy is that it is not conducting under a certain negative bias range from −0.5 to 0, but a turn-on operation in other negative states. That is to say, as for MAPbI_3_, −0.5 bias is a threshold value and the current can be conducted only beyond this threshold. Thus, based on this property, threshold voltage can be set for security under the application of some electronic security locks. As shown in Fig. 3(b)_,_ MAPbBr_3_ device has similar diode character like MAPbI_3_, which has on-state in a certain negative bias and off-state in the whole positive range. But at the on-state region, with the increase of the negative bias, the current of the MAPbBr_3_ device increases continuously and the strong NDR effect would disappear.

When MAPbI_3_ and MAPbBr_3_ are inserted into the silicon carbide nanotube, the value of the threshold voltage shifts a bit to the right, meaning that silicon carbide nanotube probably regulate the diode activity of MAPbX_3_ (X = I, Br), as shown in Fig. 3(c). The grey line represents MAPbI_3_ encapsulated in silicon carbide nanotube while the orange line represents the MAPbBr_3_ encapsulated in silicon carbide nanotube. It can be obviously seen that these two composite structures hold on-state in a certain negative bias and off-state in the whole positive range as exhibited by the individual MAPbX_3_ (X = I, Br) device. Importantly, NDR effect appears on the operating region of this MAPbI_3_-SiCNT device whose maximum peak-valley ratio (PVR) is 3.03 (28.8/9.5) and on-off ratio is 720 (28.8/0.04). These two parameters manifest that the diode performance of MAPbI_3_ becomes advanced when encapsulated in the silicon carbide nanotube.

There is another important observation after we further encapsulate MAPbI_3_ into carbon nanotube (CNT), as illustrated in Fig. 3(d). Some novel and interesting things have come up that it emerges current throughout positive voltage range different from MAPbX_3_ and MAPbX_3_-SiCNT. And MAPbI_3_-CNT sustains open-up feature in negative bias, which begins operating beyond −1.4 V. Nevertheless, MAPbBr_3_-CNT displays transmission characteristic of traditional electronic devices. What counts is that it arises two platforms on [−1 V, 0 V] and [0.5 V, 1 V] respectively.

**Figure 4:**
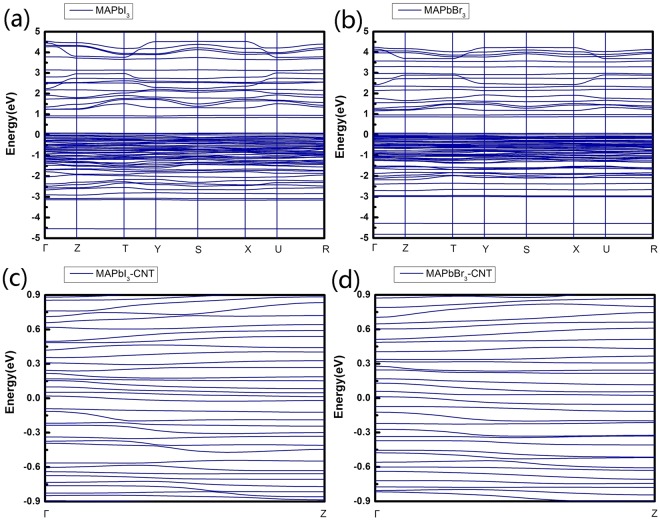
The band structure of (**a**) orthorhombic CH_3_NH_3_PbI_3_ and (**b**) CH_3_NH_3_PbBr_3_ crystals along the high-symmetry lines in the first Brillouin zone. The band structure of (**c**) CH_3_NH_3_PbI_3_ and (**d**) CH_3_NH_3_PbBr_3_ encapsulated in CNT. Γ and Z in the band structures correspond to the (0, 0, 0) and (0, 0, 0.5) k-points, respectively, in the Brillouin-zone.

The band structures of orthorhombic MAPbX_3_ (X = I, Br) calculated by the PBE/GGA functional are shown in Fig. 4(a,b). Our results are successfully match the previous theoretical value with the same function employed^31^. It can be seen that MAPbI_3_ and MAPbBr_3_ have the similar band structure because they have the same lattice constants. This phenomenon also appears in the research by Mosconi *et al*.^41^. There are obvious band gap between the VBM and CBM, showing a typical semiconductor nature for both MAPbI_3_ and MAPbBr_3_. This also make it rational for their semiconductive current-voltage characteristic curves. We also calculate the band structures when MAPbX_3_ (X = I, Br) encapsulated in CNT as shown in Fig. 4(c,d). The band gap vanishes and only one subband for MAPbI_3_-CNT and for MAPbBr_3_-CNT passes through the E_f_, respectively, showing a metal characteristic. These also correspond to their I-V curves which have both forward current and reverse current, distinguishing from that of MAPbX_3_ which has semiconductor diode rectifying.

**Figure 5:**
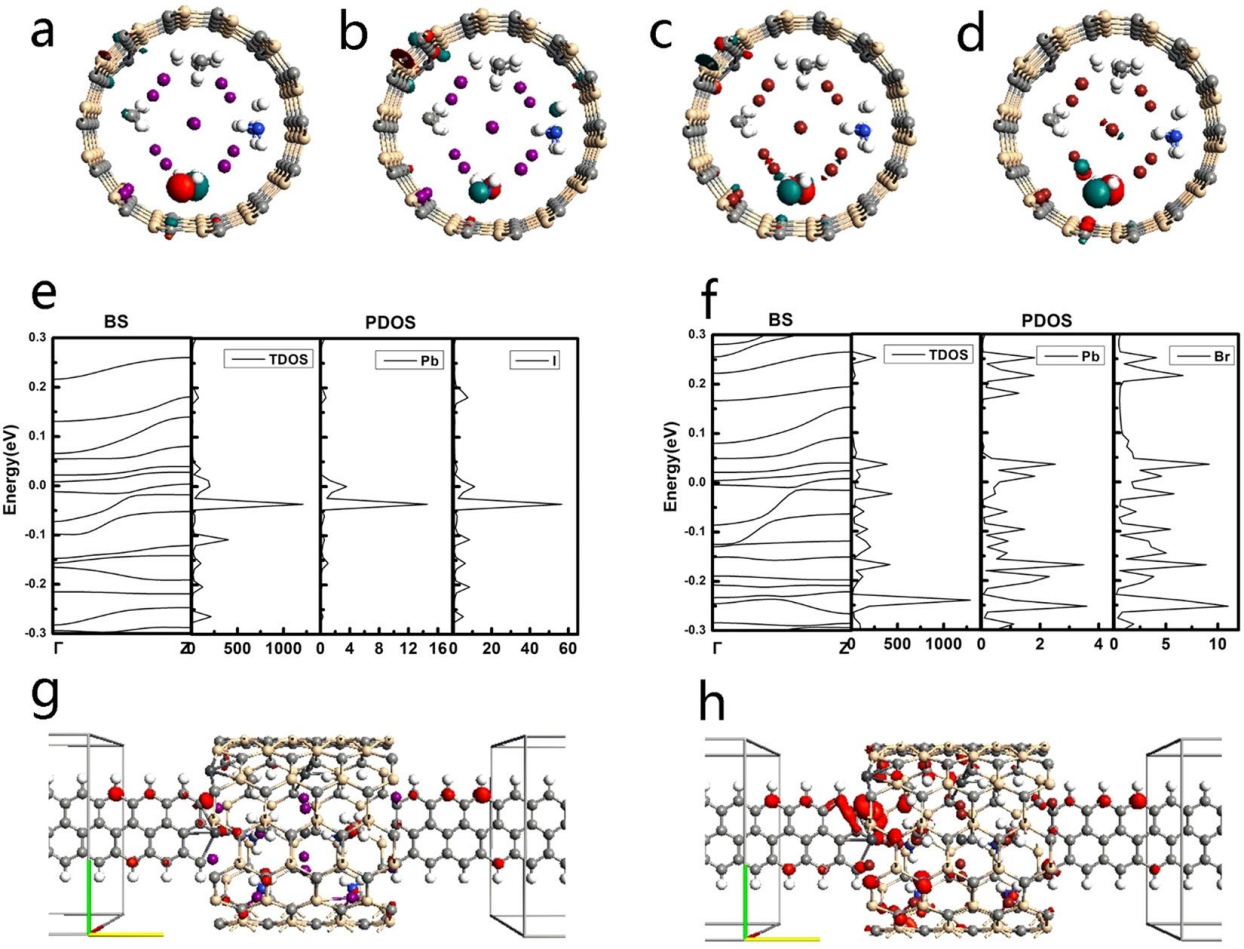
The electronic properties for MAPbX_3_-SiCNT devices. (**a**,**b**) The Bloch sates at the HOVBM and LUCBM of MAPbI_3_-SiCNT, respectively. (**c**,**d**) The Bloch sates at the HOVBM and LUCBM of MAPbBr_3_-SiCNT, respectively. (**e**,**f**) The band structure (BS), total density of states (DOS), and projected density of states (PDOS) for MAPbI_3_-SiCNT and MAPbBr_3_-SiCNT, respectively. Γ and Z in the band structures correspond to the (0, 0, 0) and (0, 0, 0.5) k-points, respectively, in the Brillouin-zone. (**g**,**h**) The local density of states (LDOS) for MAPbI_3_-SiCNT and MAPbBr_3_-SiCNT, respectively. The colors represent the phase. The isovalue is 0.1.

By moving to analyze the electronic structures of these devices, we show the density of states (DOS) and projected density of states (PDOS) as well as the band structure (BS) in Fig. 5. The PDOS is the DOS projected onto the Pb and halide atoms. After encapsulated in a SiC nanotube, bands of MAPbI3 move down and up leading to semiconductor behavior with a very narrowed gap of 16 meV. And MAPbBr_3_ has further narrowing gap after encapsulated in the SiC nanotube like MAPbI_3_. The Bloch states at the highest occupied valence band maximum (HOVBM) and the Bloch states at the lowest unoccupied conductance band minimum (LUCBM) of device MAPbI_3_-SiCNT are shown in Fig. 5(a,b). Both the HOVBM and LUCBM are located at the Γ point in the Brillouin zone, illustrating it has the feature of a semiconductor with direct band gap. As shown in Fig. 5(a–d), both the HOVBM and LUCBM of the MAPbX_3_-SiCNT corresponds to the bottom of MAPbX_3_ part localized at N atoms mainly. And the LUCBM of the MAPbI_3_-SiCNT is also localized at SiCNT partially while the HOVBM is hardly localized at SiCNT. These two corresponding Bloch states at the Γ point indicate that only the conductance band is induced by the hybridization of SiCNT and the valence band actually results from the states of the N atoms of CH_3_NH_3_^+^ cations. This result is against the result from Wang *et al*.^31^ who pointed out that organic CH_3_NH_3_^+^ cations made little contribution to the bands of CH_3_NH_3_PbI_3_ around the Fermi energy level. The SiCNT is a main factor contributing to these two results. However, the MAPbBr_3_-SiCNT has the opposite case that only the HOVBM localized at SiCNT as shown in Fig. 5(c,d), indicating its valence band is induced by the hybridization of SiCNT and the conductance band results from both the states of the N atoms of CH_3_NH_3_^+^ cations and the hybridization of SiCNT. The BS, TDOS and PDOS for MAPbX_3_-SiC are manifested in Fig. 5(e,f). The PDOS shows that the DOS originates mainly from the contribution of halide atoms rather than Pb atoms, more precisely, it results from their unsaturated p orbitals. That is to say, the chemistry modification of I/Br atoms can tune the electronic phenomenon of MAPbX_3_-SiCNT. Furthermore, we can see that PDOS peaks for Pb and X (X = I, Br) atoms can align very well, which indicates that a orbital hybridization and a strong bonding have been established among these atoms. Interestingly, the PDOS peaks of I atoms in the bottom conductance bands are stronger and sharper than those of Br atoms in the top valence bands. The PDOS images of I and Br atoms support the argument that these two kinds of halide atoms are chemically non-equivalent though MAPbI_3_-SiCNT and MAPbBr_3_-SiCNT have the same lattice structure. The different PDOS images of I and Br atoms indicate a stronger bonding between Pb and I atoms in MAPbI_3_-SiCNT than that between Pb and Br atoms in MAPbBr_3_-SiCNT. This result is different from that of MAPbX_3_ without SiCNT^41^.

**Figure 6:**
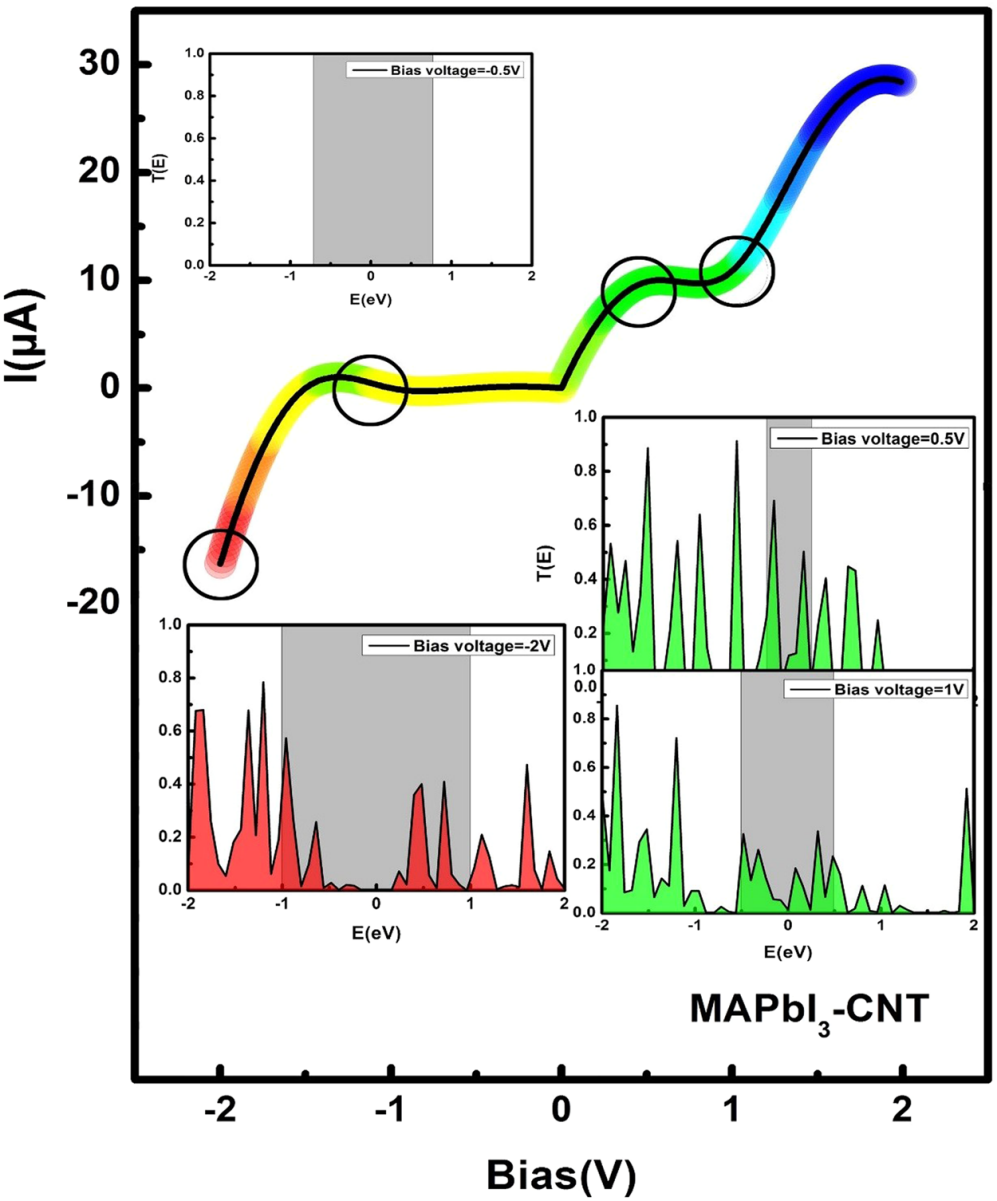
I–V curve of the MAPbI_3_-CNT device. The four typical points are labeled by circles. The inserts are transmission spectra under a bias of −2, −1.0, 0.5, and 1 V, respectively. The shaded area represents the bias window.

To explore origin of the above current-voltage characteristic, we further analyze the transmission spectra of MAPbI_3_-CNT, which is depicted in Fig. 6. The current through devices is inseparable from the transmission coefficients of devices since the current here is calculated by the Landauer-Büttiker formula. In this work, the average Fermi level, which is the average chemical potential of left and right electrodes, is set as zero. We define and discuss NDR effect follow a classical mode proposed by Shen *et al*.^42^ who employed the same simulation method and software as us. The colormap shows the current value, for example, red color stands for I = −16 μA and green color represents I = 6 μA. We select four typical points which are pointed by black circles to analyze their transmission spectra. The shaded area at every insert transmission spectra represents the energy interval of the chemical potential from the left to the right electrode. The transmission peaks within the bias window are important since they mainly contribute to the current. It is observed that values of all transmission peaks do not exceed 1, which illustrates all transmission spectra are contributed by single channel. In this work, when negative bias voltage rises to 2 V, the MAPbI_3_-CNT device has the maximum reverse current of almost 16 μA. At this moment, the bias window in transmission spectra at V = −2 V has four significant transmission peaks, elucidating the existence of transport. This result also identifies with conducting state of MAPbI_3_-CNT device. Furthermore, this device has non-conducting state in the region of [−1, 0]. To analyze this domain, we select the center dot at −0.5. From the corresponding transmission spectrum, we can see that there is no electronic transport regardless of whether at bias window nor in the whole range. When applied the positive voltage, the current increases at the beginning and then begins to decrease at V = 0.5 V and finally increases again continuously. We define the two inflection points (0.5 V, 1 V) as peak and valley respectively, which is not consistent with ohm’s law called NDR effect. It is obvious that the peaks in the 0.5 bias window are enhanced when compared to peaks in the 1 bias. Other MAPbX_3_ devices have the similar reasons.

## Discussion

In conclusion, DFT calculations have provided a new idea that MAPbX_3_ (X = I, Br) can act well as diodes and its behavior can be modulated by encapsulation of the silicon carbide/carbon nanotube (SiCNT/CNT). It can be observed that MAPbX_3_ models display on-state in the negative bias and off-state in the positive range. And the diode activity of MAPbX_3_ changes after inserted into SiCNTs. It could be applied in some electronic security locks, based on the character that they only conduct in a certain bias. In addition, when MAPbX_3_ are inserted into CNTs, it emerges current throughout positive voltage range different from MAPbX_3_ and MAPbX_3_-SiCNT. Origin of their diode behavior is interpreted by their transmission spectra and density of states. The electronic structures are also discussed. This represents an important step in designing new diodes for high-efficiency electronic components.

## Methods

In this work, we employ the first-principles density functional theory (DFT) in combination with the Non-equilibrium Green Function (NEGF) method as implemented in Atomistic ToolKit (ATK) software package to calculate transport properties. The device is divided into two parts, that is, the electrode and the channel. The source and drain electrodes are composed of graphene nanoribbons (GNRs), between which is the electron transfer channel (MAPbX_3_), as shown in Fig. 2. The mesh cutoff for the electrostatic potentials is 75 Ha. Double-zeta single polarized basis sets of local numerical orbitals and generalized gradient approximations (GGA) for exchange correlation potentials are used. K samples in the x, y, and z directions are 3, 3, and 50, respectively. The standard of convergence of the total energy is set to 10^−5^ eV.

The current I is calculated by Landauer formula when coherent transport of electrons occurs between left and right electrodes with Fermi levels *μ*_*L*_ and *μ*_*R*_ through the central scattering region^43^:

$$I=\frac{2e}{h}{\int }_{-\infty }^{\infty }dE({\rm{T}}({\rm{E}},{\rm{V}})({f}_{1}(E)-{f}_{2}(E)))$$

where *T*(*E*, *V*) is the transmission probability of electrons from left to right region, *f*_1,2_(*E*) is the Fermi-Dirac distribution function of the source and drain respectively, e and h are the electron charge and Planck constant, respectively.

## References

[1] Kojima A, Teshima K, Shirai Y, Miyasaka T (2009). Organometal halide perovskites as visible-light sensitizers for photovoltaic cells. J. Am. Chem. Soc..

[2] Manser JS, Saidaminov MI, Christians JA, Bakr OM, Kamat PV (2016). Making and Breaking of Lead Halide Perovskites. Acc. Chem. Res..

[3] Saparov B, Mitzi DB (2016). Organic-Inorganic Perovskites: Structural Versatility for Functional Materials Design. Chem. Rev..

[4] Sutherland BR, Sargent EH (2016). Perovskite photonic sources. Nat. Photonics.

[5] Yusoff AR, Nazeeruddin MK (2014). Organohalide Lead Perovskites for Photovoltaic Applications. J. Phys. Chem. Lett..

[6] Grätzel C, Zakeeruddin SM (2013). Recent trends in mesoscopic solar cells based on molecular and nanopigment light harvesters. Mater. Today.

[7] Wei H (2016). Sensitive X-ray detectors made of methylammonium lead tribromide perovskite single crystals. Nat. Photonics.

[8] Deschler F (2014). High Photoluminescence Efficiency and Optically Pumped Lasing in Solution-Processed Mixed Halide Perovskite Semiconductors. J. Phys. Chem. Lett..

[9] Tan ZK (2014). Bright light-emitting diodes based on organometal halide perovskite. Nat. Nanotechnol..

[10] Ho-Baillie A, Snaith HJ, Green MA (2014). The emergence of perovskite solar cells. Nat. Photonics.

[11] Kim HS, Sang HI, Park NG (2014). Organolead Halide Perovskite: New Horizons in Solar Cell Research. J. Phys. Chem. C.

[12] Mcmeekin DP (2016). A mixed-cation lead mixed-halide perovskite absorber for tandem solar cells. Science.

[13] Park NG (2013). Organometal Perovskite Light Absorbers Toward a 20% Efficiency Low-Cost Solid-State Mesoscopic Solar Cell. J. Phys. Chem. Lett..

[14] Snaith HJ (2013). Perovskites: The Emergence of a New Era for Low-Cost, High-Efficiency Solar Cells. J. Phys. Chem. Lett..

[15] Wasylishen RE, Knop O, Macdonald JB (1985). Cation rotation in methylammonium lead halides. Solid State Commun..

[16] Swainson IP, Hammond RP, Soullière C, Knop O, Massa W (2003). Phase transitions in the perovskite methylammonium lead bromide, CH_3_ND_3_PbBr_3_. J. Solid State Chem..

[17] Kitazawa N, Watanabe Y, Nakamura Y (2002). Optical properties of CH_3_NH_3_PbX_3_ (X = halogen) and their mixed-halide crystals. J. Mater. Sci..

[18] Tanaka K (2003). Comparative study on the excitons in lead-halide-based perovskite-type crystals CH_3_NH_3_PbBr_3_ CH_3_NH_3_PbI_3_. Solid State Commun..

[19] Poglitsch A, Weber D (1987). Dynamic disorder in methylammoniumtrihalogenoplumbates (II) observed by millimeter‐wave spectroscopy. J. Chem. Phys..

[20] Onoda-Yamamuro N, Matsuo T, Suga H (1990). Calorimetric and IR spectroscopic studies of phase transitions in methylammonium trihalogenoplumbates. J. Phys. Chem. Solids.

[21] Gordillo, G., Otálora, C. A. & Reinoso, M. A. Trap center study in hybrid organic-inorganic perovskite using thermally stimulated current (TSC) analysis. *J. Appl. Phys*. **122** (2017).

[22] Zhang F (2015). Brightly Luminescent and Color-Tunable Colloidal CH_3_NH_3_PbX_3_ (X = Br, I, Cl) Quantum Dots: Potential Alternatives for Display Technology. Acs Nano.

[23] Vybornyi O, Yakunin S, Kovalenko MV (2015). Polar-solvent-free colloidal synthesis of highly luminescent alkylammonium lead halide perovskite nanocrystals. Nanoscale.

[24] Shamsi J (2016). N-Methylformamide as a Source of Methylammonium Ions in the Synthesis of Lead Halide Perovskite Nanocrystals and Bulk Crystals. ACS Energy Lett..

[25] Gil-Escrig L, Miquel-Sempere A, Sessolo M, Bolink HJ (2015). Mixed Iodide-Bromide Methylammonium Lead Perovskite-based Diodes for Light Emission and Photovoltaics. J. Phys. Chem. Lett..

[26] Jang DM (2015). Reversible Halide Exchange Reaction of Organometal Trihalide Perovskite Colloidal Nanocrystals for Full-Range Band Gap Tuning. Nano Lett..

[27] Ling Y (2016). Bright Light‐Emitting Diodes Based on Organometal Halide Perovskite Nanoplatelets. Adv. Mater..

[28] Pathak Sandeep, Sakai Nobuya, Wisnivesky Rocca Rivarola Florencia, Stranks Samuel D., Liu Jiewei, Eperon Giles E., Ducati Caterina, Wojciechowski Konrad, Griffiths James T., Haghighirad Amir Abbas, Pellaroque Alba, Friend Richard H., Snaith Henry J. (2015). Perovskite Crystals for Tunable White Light Emission. Chemistry of Materials.

[29] Yuan Z, Shu Y, Xin Y, Ma B (2016). Highly luminescent nanoscale quasi-2D layered lead bromide perovskites with tunable emissions. Chem. Commun..

[30] Arora, N. *et al*. Perovskite solar cells with CuSCN hole extraction layers yield stabilized efficiencies greater than 20. *Science*, eaam5655 (2017). 10.1126/science.aam5655 28971968

[31] Wang Y (2014). Density functional theory analysis of structural and electronic properties of orthorhombic perovskite CH_3_NH_3_PbI_3_. Phys. Chem. Chem. Phys..

[32] Feng J, Xiao B (2014). Crystal Structures, Optical Properties, and Effective Mass Tensors of CH_3_NH_3_PbX_3_ (X = I and Br) Phases Predicted from HSE06. J. Phys. Chem. Lett..

[33] Wang D, Wright M, Elumalai NK, Uddin A (2016). Stability of perovskite solar cells. Sol. Energy Mater. Sol. Cells.

[34] Sarswat PK, Free ML (2015). Long-term Stability of Mixed Perovskites. Mrs Online Proceedings Library Archive.

[35] Sn H (2014). Carbon Nanotube/Polymer Composites as a Highly Stable Hole Collection Layer in Perovskite Solar Cells. Nano Lett..

[36] Muhulet Alexandru, Miculescu Florin, Voicu Stefan Ioan, Schütt Fabian, Thakur Vijay Kumar, Mishra Yogendra Kumar (2018). Fundamentals and scopes of doped carbon nanotubes towards energy and biosensing applications. Materials Today Energy.

[37] Pitkänen, O. *et al*. On-chip integrated vertically aligned carbon nanotube based super- and pseudocapacitors. *Sci*. *Rep*. **7** (2017). 10.1038/s41598-017-16604-x PMC5707404 29185493

[38] Smith BW, Monthioux M, Luzzi DE (1999). Carbon nanotube encapsulated fullerenes: a unique class of hybrid materials. Chem. Phys. Lett..

[39] Sarswat PK, Smith YR, Free ML, Misra M (2015). Duality in Resistance Switching Behavior of TiO_2_ -Cu_2_ZnSnS_4_ Device. J. Solid State Sci. Technol..

[40] Tang W (2010). Memory Effect and Negative Differential Resistance by Electrode Induced Two Dimensional Single Electron Tunneling in Molecular and Organic Electronic Devices. Adv. Mater..

[41] Mosconi E, Umari P, De Angelis F (2016). Electronic and optical properties of MAPbX_3_ perovskites (X = I, Br, Cl): a unified DFT and GW theoretical analysis. Phys. Chem. Chem. Phys..

[42] Shen L (2010). Electron transport properties of atomic carbon nanowires between graphene electrodes. J. Am. Chem. Soc..

[43] Landauer R (1996). Spatial variation of currents and fields due to localized scatterers in metallic conduction (and comment). J. Math. Phys..

